# Announcement: Third International Symposium on Metal Ions in Biology & Medicine

**DOI:** 10.1155/MBD.1994.81

**Published:** 1994


					THIRD INTERNATIONAL SYMPOSIUM
ON METAL IONS IN BIOLOGY & MEDICINE
MAY 17-21, 1994
International Organizing Committee:
Co-Chairman: Neil A. Littlefield Co-Chairman: Lionel A. Poirier
Pllilippe Collery, James E. Crook, John N. Hathcock, Kazimierz S. Kasprzak, David G. Longfellow,
Richard L. Nelson, F. William Sunderman, F. William Sunderman, Jr., Michael P. Waalkes
Registration for the meeting will cover meeting attendance and-a banquet, which will be held on
Thursday evening, and a copy of the published proceedings. Hotel registration and meals will be
separate. Cost of registration will be $1 5;0.00 American before February 15th and $1 75.00
American after that date.
All inquiries relating to the Symposium should be addressed to either:
Neil A. Littlefield or Lionel A. Poirier
NCTR, 3900 NCTR Drive, Jefferson, Arkansas 72079
Tel: (501) 543-7524 Fax: (501) 543-7136
Meeting site and hotel accommodations:
Le Chateau Champlain, 1, Place du Canada, Montreal (Quebec), Canada H3B 4C9
Tel.: (514) 878-9000- Fax: (514) 878-6761
A block of guest rooms have been reserved for Symposium participants at special rates at Le
Chateau Champlain. The hotel is 19 miles from Dorval Airport with airport shuttle ($8.50 one way)
and taxi ($19-23) service available. The hotel is 45 miles from Mirabel Airport (shuttle $12; taxi $60).
Room rates at the Le Chateau Champlain will be $100.00/day (Canadian).
Negotiations are in progress to publish the proceedings of the Symposium. They will be available
on the date of the Symposium and will be published as the 3rd volume of Metal Ions in Biology
and Medicine
Special events and tours will be organized. There is an abundance of opportunities for sightseeing
and tours will be provided for attendees and their spouses. In addition, a children's program and
child care will be available.
The deadline for submission of abstracts is January 15,1994. A 5-page camera-ready
manuscript will be required for presenter which will be published in the Symposium proceedings.
SymposiumTopics
The following topics will be discussed in relation to essential metal ions, toxic metal ions, and metal
interactions:
Biological/molecular interactions. Chemotherapy. Aging. Nutrition. Carcinogenesis. Immunology.
Epidemiology. Toxicology. Cardiovascular diseases. Reproductive toxicity and teratogenesis.
Neurotoxicity. Pathogenicity of metal internal protheses (including dental). Chemistry/Biochemistry.
This Symposium will address the role of metal ions in areas of biochemical, biological, and medical
sciences. The meetings will be of practical usefulness to those in basic and applied research,
clinical scientists, and workers in allied medical fields, toxicologists, and microbiologists.
The meetings will be bilingual in English and in French with translation available.

				

